# How Dogs Perceive Humans and How Humans Should Treat Their Pet Dogs: Linking Cognition With Ethics

**DOI:** 10.3389/fpsyg.2020.584037

**Published:** 2020-12-16

**Authors:** Judith Benz-Schwarzburg, Susana Monsó, Ludwig Huber

**Affiliations:** ^1^Unit of Ethics and Human-Animal Studies, Messerli Research Institute, Vetmeduni Vienna, University of Vienna, Medical University of Vienna, Vienna, Austria; ^2^Unit of Comparative Cognition, Messerli Research Institute, Vetmeduni Vienna, University of Vienna, Medical University of Vienna, Vienna, Austria

**Keywords:** animal cognition, social cognition, animal ethics, human-animal interactions, positive duties, trust

## Abstract

Humans interact with animals in numerous ways and on numerous levels. We are indeed living in an “animal”s world,’ in the sense that our lives are very much intertwined with the lives of animals. This also means that animals, like those dogs we commonly refer to as our pets, are living in a “human’s world” in the sense that it is us, not them, who, to a large degree, define and manage the interactions we have with them. In this sense, the human-animal relationship is nothing we should romanticize: it comes with clear power relations and thus with a set of responsibilities on the side of those who exercise this power. This holds, despite the fact that we like to think about our dogs as human’s best friend. Dogs have been part of human societies for longer than any other domestic species. Like no other species they exemplify the role of companion animals. Relationships with pet dogs are both very widespread and very intense, often leading to strong attachments between owners or caregivers and animals and to a treatment of these dogs as family members or even children. But how does this relationship look from the dogs’ perspective? How do they perceive the humans they engage with? What responsibilities and duties arise from the kind of mutual understanding, attachment, and the supposedly “special” bonds we form with them? Are there ethical implications, maybe even ethical implications beyond animal welfare? The past decades have seen an upsurge of research from comparative cognition on pet dogs’ cognitive and social skills, especially in comparison with and reference to humans. We will therefore set our discussion about the nature and ethical dimensions of the human–dog relationship against the background of the current empirical knowledge on dog (social) cognition. This allows us to analyze the human–dog relationship by applying an interdisciplinary approach that starts from the perspective of the dog to ultimately inform the perspective of humans. It is our aim to thereby identify ethical dimensions of the human–dog relationship that have been overlooked so far.

## Introduction

The question of how dogs perceive us humans is important for several reasons, both from the perspective of biologists as well as animal ethicists. First, an enduring topic of animal behavior and animal cognition research is how animals adapt to their social environment, how they cope with the challenges of dynamic relationships among group members, and especially how they achieve a balance between competition and cooperation. Complex social life has been proposed as one of the main driving forces in the evolution of higher cognitive abilities in humans and non-human animals (Humphrey, 1976; Dunbar, 1998).

Secondly, while evolution has equipped species with the appropriate cognitive tools to engage in sophisticated social interactions during foraging and conflict management, including the formation of valuable relationships (social bonds), it is less clear how species became able to deal with heterospecifics with whom they live in close interaction, i.e., not simply as prey or predator. This is the case in at least two domains, in urban species and in domesticated species. In the latter domain, dogs have been considered as the species that formed the closest bonds with humans. So how was it possible for these animals to engage in such close interactions with humans, who are members of a different species, with a different anatomy, physiology, including different sensory modalities, behavior, and cognition?

While the first two reasons might inspire cognitive biologists who address topics in animal behavior and evolution to investigate dogs’ perspective on the human–dog-relationship, animal ethicists might find additional reasons why the question of how dogs perceive humans is important. This is because the relationship between humans and dogs is characterized by a clear dominance hierarchy, not only during the process of domestication, but also during the individual life of the dog. This only gives us an ethical reason why to consider the human–dog-relationship but also a reason why to consider it differently than relationships that are not characterized in such a way. Humans have domesticated dogs, not vice versa, mainly to exploit them for their own benefit, as assistants during hunting, as guardians of their homes, or as companions. More recently, we have added other tasks and purposes that cover a very wide range of different contexts. We use dogs as testing devices in labs, as search (and rescue) animals (when looking for missing persons as much as when looking for rare truffles), as therapists in animal-assisted therapies, dance partners in dog dancing, hair models in dog grooming, or influencers in social media, just to name a few. The multitude of interactions and contexts in which we use them, of course, has produced a number of welfare issues and, as we are going to argue, ethical issues beyond welfare. While ethical debates have convincingly pointed to human responsibilities for example in the case of farm animals and lab animals, companion animals are often not so clearly seen as animals which we “use,” objectify, or instrumentalize, maybe because the term “companion” indicates to some degree a mutual relationship rather than an exploitative one. But how, in fact, do dogs experience this relationship? How do they perceive the humans they engage with? Have they indeed specifically adapted to interact and form “special” bonds with humans as the Domestication Hypothesis (see our section on Effects of Domestication) suggests? We assume that part of the answer to these questions can be found in the growing evidence for dogs’ special skills to perceive and understand us.

The structure of this paper is as follows. In a first step, we will discuss insights from the dog’s domestication history and from empirical studies on their (social) cognition to illustrate how dogs perceive us, and consequently sketch the nature of our relationship with them. In a second step, we will assess what ethical responsibilities arise from the characteristics of the human–dog relationship. Should we profoundly reevaluate some ways we use dogs, and enrich the narrative of dogs as “companions” and “man’s best friend” with some ethical considerations that are indeed more demanding? Our methodology thus utilizes the results from current debates in dog social cognition to evaluate the human–dog relationship from a critical, ethical perspective. Our aim is to show by means of such an interdisciplinary investigation in what ways our current knowledge about dog domestication and dog social cognition can and should inform our treatment of these animals. For our discussion of the empirical evidence, we have picked three areas of dog social cognition where we find a substantial amount of studies. Our selection thus mirrors the general interest of the research community. However, the community might be neglecting other possible abilities in dogs due to a lack of interest in them, a publication bias towards positive results, flawed study designs or other reasons. We will come back to this in our ethical discussion, since what we do not know about dogs might be relevant to the treatment that we owe them. While in this paper we will restrict our discussion of ethical implications to the kinds of studies available, other, more profound ethical implications might lie ahead, once cognition research broadens its focus.

## Characterizing the Human–Dog Relationship: Biological Perspectives

In this section, we will investigate the characteristics of the human–dog relationship by following the decisive question of how dogs adapt to the human environment. We will turn our attention to the latest research results from the fields of animal cognition and behavior. The default assumption is that dogs’ skills are firmly based on some general canine abilities of intraspecies communication plus a combination of phylogenetic and ontogenetic abilities of interspecies communication. The latter ones have emerged from domestication and individual social and cognitive development (Huber, 2016). Both kinds of developmental factors have contributed to the success of dogs living among and with humans, including their adoption of the numerous roles humans give to them.

### Effects of Domestication: New Skills or Special Sensitivity?

For thousands of years humans have changed the morphology, physiology, and behavior of dogs through selective breeding. Canines were the earliest domesticated animal, a process that started somewhere between 15,000 and 30,000 years ago, most likely when gray wolves began scavenging around human settlements. Dog experts differ on how active role humans played in the next step, but eventually the relationship became a mutual one, as we began employing dogs for hunting, guarding, and companionship.^1^

It is, however, still an open question to what extent the three kinds of cognitive and communicative adaptations – of the wolf, the dog, and the human companion (pet) – contribute to this extraordinary achievement. It is furthermore disputable if the outcome of these different developments is a new skill or rather a special sensitivity. In addition, we may distinguish not only between phylogenetic and ontogenetic routes, but also between construction and inflection (Heyes, 2003), to overcome the simplistic dichotomy of nature vs. nurture. One cautionary application of the multiple routes framework would be to assume that dogs have acquired a special sensitivity towards human gestures, speech, and behavior as a phylogenetic inflection through human selection over many thousands of years. This sensitivity is not a new cognitive or sensory mechanism, but the result of a selection biasing the input.

Since the time dogs became a special focus of ethology and comparative cognition research, the so-called Domestication Hypothesis has dominated the debate about the special skills of dogs (Hare et al., 2002; Topál et al., 2009; Miklósi and Topál, 2013). It has been assumed that dogs have been selected to cooperate and communicate with humans during domestication and, thus, evolved some genetic predispositions allowing them to develop skills shared with humans. Accordingly, it has been suggested that, in a unique way, domestication has equipped dogs with two abilities necessary for cooperative problem solving – namely social tolerance and social attentiveness, which enable them to adjust their behavior to that of their human partners (Ostojic and Clayton, 2014).

Empirical support for the Domestication Hypothesis has been sought by comparing dogs and wolves. Several of the early comparisons have indeed found profound differences between domesticated forms and their wild ancestors (i.e., the closest wild-living relatives) in the way they communicate and cooperate with humans, for instance in following human gestures, as well as in their capacities for social tolerance and social attentiveness. It has been proposed that dogs have been selected for tamer temperament and for reduced fear and aggression, which allows a potential partner to come close even around food, which in turn explains the higher success of dogs in cooperative and communicative interactions with humans in comparison to wolves (Hare and Tomasello, 2005).

Apart from social tolerance, cooperation with humans and learning from humans are facilitated by a high degree of social attentiveness. Cooperation requires that the partners pay sufficient attention to each other in order to adjust or synchronize their behavior, and social learning requires paying attention to the demonstrator’s actions and the context in which they are executed (Huber et al., 2009). Attentiveness towards potential partners varies not only according to the tasks, but at least in the human–dog case, it crucially depends on the relationship between the partners (Range et al., 2007; Horn et al., 2013). Dogs have proven successful in several tasks that are thought to require high attention towards humans, such as experiments on social learning (Kubinyi et al., 2003; Topál et al., 2006; Huber et al., 2009, 2014; Range et al., 2011; Fugazza and Miklósi, 2014), social referencing (Merola et al., 2012a,b), communication (Virányi et al., 2004; Schwab and Huber, 2006; Udell and Wynne, 2008; Dorey et al., 2009; Kaminski et al., 2012), responding to unequal rewards (Range et al., 2009), and cooperation (Naderi et al., 2001; Bräuer et al., 2013; Ostojic and Clayton, 2014).

Another line of evidence for the differences between dogs and wolves comes from pointing studies. Young dogs follow human pointing better and look at humans more readily than human-raised wolves (Miklósi et al., 2003; Gácsi et al., 2009). This led researchers to propose that dogs have developed increased social attentiveness compared to wolves and, thus, can achieve more complex forms of dog–human communication and cooperation than wolves (Miklósi et al., 2003; Virányi et al., 2008).

However, as most of the studies compared the animals’ interactions only with humans (Hare et al., 2002; Miklósi et al., 2003; Topál et al., 2005; Udell and Wynne, 2008; Virányi et al., 2008; Gácsi et al., 2009; Udell et al., 2011), it remained unclear whether the differences between dogs and wolves reflect mere differences in the readiness of dogs and wolves to interact with humans or more fundamental differences regarding intraspecific cooperation. Indeed, experiments at the Wolf Science Center in Austria have shown that (hand-raised) wolves pay as much attention to human partners as dogs do and that these wolves can even outperform dogs in learning from observation of a conspecific, indicating the high social attentiveness of the species (Range and Virányi, 2013, 2014). Accordingly, the so-called Canine Cooperation Hypothesis postulates that dog-human cooperation evolved on the basis of wolf–wolf cooperation and that no additional selection for social attentiveness and tolerance was necessary to allow for dog-human cooperation to evolve (Range and Virányi, 2014, 2015; Virányi and Range, 2014). Rather than tolerance, domestication may have led to reduced fear of humans, which is supported by the fact that dogs need less intensive socialization than wolves to avoid fear of humans (Scott and Fuller, 1965; Klinghammer and Goodmann, 1987). If dogs are less fearful of humans and more comfortable around them than wolves, they would have gained advantages from witnessing human actions (even without being more attentive), and from sooner engaging in interactions with humans.

According to the Canine Cooperation Hypothesis, the high social attentiveness, tolerance, and presumable cooperativeness of wolves provided a good basis for dog–human cooperation to evolve during domestication. In addition, some relevant features in sociability and cooperativeness are shared by wolves and humans and thus have probably facilitated the domestication of dogs (Clutton-Brock, 1984; Schleidt, 1998). However, dogs are not only specifically sensitive to humans because of the domestication history of their species and the evolutionary baggage that has been passed down to them from their wild ancestors, the wolves. They are also what they are because each of them trains their outstanding sensitivity towards humans on an individual, ontogenetic level.

### Individual Development

Despite being equipped by evolution with skills and propensities to adapt to humans by showing high levels of social tolerance and attentiveness, dogs need to individually learn much about their heterospecific partners in order to establish and maintain firm individualized relationships. During their life in the human household as pets or companions, they have ample opportunities to do so. Family dogs live in close day-to-day contact with humans and can therefore collect an enormous amount of experience. Research from the last decades has sought to understand how dogs perceive elements of their environment, learn about it, and use this knowledge to make informed decisions about proper behavior (Huber, 2016). Their skills in face processing, behavior reading, observational learning, and perspective taking play a crucial role here (for reviews, see Bensky et al., 2013; Kaminski and Marshall-Pescini, 2014; Lea and Osthaus, 2018). In what follows, we will summarize recent findings on dogs’ understanding of human emotions, gestures, and actions.

#### Understanding Human Emotions: How Dogs Read Our Faces and Listen to Our Voices

Interspecies emotional communication is in part facilitated by chemosignals (D’Aniello et al., 2018), but, faces are in addition an important visual category for many species because they provide a rich source of perceptual cues, including many idiosyncratic features, and thus facilitate important discriminations. In the specific case of dogs, it has been suggested that their increased readiness to look at the human face provides a basis for complex forms of dog-human communication (Miklósi et al., 2003). By monitoring human faces, dogs seem to obtain important social information, ranging from communicative gestures to attentive states (Schwab and Huber, 2006; Kaminski and Nitzschner, 2013). Dogs can quickly find out what features are relevant or informative for making important decisions. They also spontaneously focus on the eyes to infer where humans attend, what they are interested in, and even what they intend to do next (see eye movement studies like for example Somppi et al., 2014).

Gaze following is present in many species, but dogs outperform even nonhuman primates in following human gaze in object choice tasks (Hare et al., 2002; Cooper et al., 2003). Like in the case of human infants, their gaze following is modulated by ostensive cueing, such as direct gaze and addressing by the person, which is evidence that it is more than simply a product of reflexive and learnt mechanisms (Téglás et al., 2012). Dogs also follow human’s gaze into distant space (Wallis et al., 2015), and they use the eyes of humans to judge their attentional state. In one study, dogs were tempted with sausages but told by the caregiver not to take them. The dogs obeyed more or less depending on the caregiver’s attention to them (Schwab and Huber, 2006). When being watched by the caregiver, dogs stayed lying down most often or for the longest time, but when the caregiver read a book, watched TV, turned her back on them, or left the room, their patience ceased. Obviously, they were using eye contact and eye orientation as cues.

Human faces provide much more information than simply looking patterns. A great number of idiosyncratic features allow humans to identify and recognize others. Would dogs also profit from this rich source of information? Could they also identify and recognize their caregiver and other familiar humans? In one study we put these questions to test and asked dogs to discriminate between their caregiver and another highly familiar person by active choice (approaching and touching; Huber et al., 2013). The task could not simply be solved on the basis of familiarity (approaching the familiar person), which is considered an easier task (Wilkinson et al., 2010), but required a fine-grained distinction of familiar people. Dogs could do so, even when they saw only the (real) face of the humans, but had difficulties when the face was only projected as a picture to a big screen. Only a minority of dogs could finally identify the caregiver on face pictures in which the outer parts of their faces were occluded with a balaclava hood. A further study confirmed the importance of human eyes for dogs, because they rely less on the nose or the mouth than on the eyes for human face discrimination (Pitteri et al., 2014). They also prefer looking at upright over inverted faces, exactly as we ourselves do (Somppi et al., 2012, 2014).

On the basis of our findings that dogs are competent enough to extract subtle, idiosyncratic features of a face in order to identify a human person, despite changes of color, hair style, make-up, jewelry, hats, etc., we went one step further and asked whether dogs may also learn from our facial expressions. It has been already shown that dogs can rely on human facial expressions when making decisions about approaching other objects (Merola et al., 2012a). However, a study in which the stimuli were photographs showing human faces with two different emotional expressions did not yield conclusive results (Nagasawa et al., 2011). Although dogs learned to discriminate between happy (smiling) faces and neutral faces of their caregiver and subsequently transferred the contingency to novel faces of unfamiliar people, it is not clear whether the dogs simply used a salient discriminatory cue, such as the visibility of teeth in the happy faces, to solve both the discrimination and the generalization task.

In the Clever Dog Lab in Vienna, we asked dogs to discriminate “hemifaces” – either the lower or the upper half of the faces – of women showing different (happy and angry) emotions. With this trick we could investigate whether dogs solve the task solely by attending to the emotional expression rather than any inadvertent cues in the presented human face (Müller et al., 2015). Given that the simple discriminatory cues in one half of the faces – such as teeth in the lower half – were absent in the other half, the authors could test the dogs’ ability to spontaneously categorize novel pictures on the sole basis of the emotional expression, provided globally and not just by local cues. Indeed, the dogs did not only manage to learn the training task, but they were also able to transfer the extracted rule to novel faces, even if they had been presented a hemiface not shown in training.

These findings provide strong evidence that dogs are able to discriminate between emotional expressions in a different species, which, compared to emotion recognition in conspecifics, is particularly challenging (cf. Parr et al., 2008). For instance, humans open their mouth and show their teeth while laughing, whereas dogs express the underlying emotions of aggression by showing their teeth. Therefore, dogs cannot rely on genetic predispositions, but need to individually learn the emotional expressions of humans. The fact that dogs could spontaneously generalize from one face half to the other without the possibility to use cues learned during training strongly supports the idea that they remembered something from their daily experiences with their caregiver or other familiar people and then used this information in the artificial laboratory environment. As they had not been explicitly trained, it seems that they had acquired the competence by latent learning.

Humans express their emotions not only visually but also their voices convey information about affects. Dogs may exploit these contingencies by extracting and integrating bimodal sensory emotional information from humans. From the combination of visual and auditory cues they may form multimodal representations. Using a cross-modal preferential looking paradigm, researchers at the University of Lincoln (United Kingdom) managed to show that dogs spontaneously combine human or dog faces with different emotional valences (happy/playful versus angry/aggressive) with a single vocalization from the same individual of the same positive or negative valence (Albuquerque et al., 2016). This result points to the possibility that dogs recognized or understood the emotional content of the human faces, not just discriminated them perceptually. Recent eye-tracking studies have supported this hypothesis (Barber et al., 2016; Somppi et al., 2016).

The ability of dogs to integrate information of humans across modalities has also been investigated by using the expectancy-violation procedure (Adachi et al., 2007). A photograph of either the caregiver’s face or an unfamiliar person’s face was presented to the dog after a vocalization was played. The vocalization used was from the same person or another person, thus matched or mismatched the image. According to the expectancy-violation logic, dogs should be surprised if the visual and auditory cues mismatch and thus look longer than when the two cues match. This is what happened. After hearing the caregiver’s voice when the face of an unfamiliar person appeared (incongruent condition), dogs exhibited extended looking, while in the case when the vocalization and face matched (i.e., came from the same person; congruent condition), the duration of their gaze was comparably briefer. These findings lend support to the hypothesis that dogs recall their caregiver’s face upon hearing the caregiver’s voice.

Taken together, there is cumulating evidence that dogs obtain social information from their experiences with humans, specifically from their facial expressions. They can recognize and remember individual humans. They understand to a significant degree what these humans attend to, what they are interested in, and what they intend to do next. They can discriminate, individually learn from, and categorize emotional expressions, and they integrate information coming from vocalizations into their understanding of humans and their emotions. Thus, they form multi-modal representations of humans and their emotions, integrating emotions, facial expressions, and vocalizations.

#### Understanding Human Gestures: How Dogs Learn to Cooperate

Due to domestication programs that had the goal of producing companions that work with or for humans, and thereby follow human commands, dogs may have acquired a special sensitivity to human gestures, speech, and behavior (Miklósi and Topál, 2013). Neither the chimpanzee, humans’ closest living relative, nor the wolf, dogs’ closest living relative, can understand and use human communicative cues as flexibly as the domestic dog (Kaminski and Nitzschner, 2013). This kind of phylogenetic enculturation that took place over thousands of years is continued and amplified in the course of their lifespan, as companion dogs collect an enormous amount of experience during their life with humans (Topál et al., 1998; Udell and Wynne, 2008, 2010; Topál and Gácsi, 2012). A prominent example of how well dogs understand humans and how eager they are to cooperate is the behavior of assistance dogs, especially for leading blind people (Naderi et al., 2001). In the latter case, information is not only provided but also accepted by both parties in the course of their joint actions. So what exactly do dogs learn about our behavior, especially about human actions that are unlikely in their species-specific action repertoire? An especially interesting group of actions are those that serve us humans to inform the dog or to guide them.

One of the best examples of dogs’ socio-cognitive skills is their ability to properly respond to human cues in a cooperative search context. Numerous studies have shown that dogs can reliably follow a set of basic human cues (e.g., distal/proximate pointing, head turns, and eye glances), as well as being adept at flexibly generalizing this behavior to relatively novel human movements (e.g., “cross-pointing,” leg pointing, gestures with reversed direction of movement, and different arm extensions; Soproni et al., 2002; Udell et al., 2008). In contrast, substituting the hand with a stick or preventing the dog from seeing the hand protruding from the body contour decreased performance, thereby pointing to the importance of the human’s hand. In addition to questions about the cognition involved in dogs’ responding to human cueing, experiments have flourished that systematically tested the contexts, the time-course, breed differences, training effects, and other aspects of this canine competence (review in Bensky et al., 2013).

Among those actions, perhaps the best studied one is the human pointing gesture. First of all, pointing by humans is a social cue, which in general is more salient or effective than non- social cues like visual markers in terms of signaling the location of something important, like food (Agnetta et al., 2000; Udell et al., 2008). In sharp contrast to apes (Herrmann and Tomasello, 2006), this ability to use human cues by dogs is more effective in cooperative contexts (Wobber and Hare, 2009) than in competitive ones (Pettersson et al., 2011).

Although so far there is no consensus among researchers about when exactly dogs become competent at understanding the pointing gesture (e.g., Dorey et al., 2010), it is obvious that individual learning is very effective. Even hand-raised adult wolves are as successful in relying on distal momentary pointing as adult pet dogs (Gácsi et al., 2009). Still, positive feedback processes (both evolutionary and epigenetic) have increased the readiness of dogs to attend to humans, providing the basis for dog-human communication. Among dogs, breeds that have been historically bred for working purposes respond to human pointing cues significantly more than breeds that have been bred for companionship (Wobber and Kaminski, 2011), and breeds that were originally bred for cooperative work (e.g., herding) performed better than those that were bred for independent work (e.g., guarding; Gácsi et al., 2009). Furthermore, those with a special training for responding to cues from a distance, like working-gun dogs, utilized a pointing cue significantly more than dogs without such training (McKinley and Sambrook, 2000). Independent of breed differences, shelter dogs are less successful than pet dogs at following a distal momentary-pointing gesture (Udell et al., 2008).^2^ Lastly, dogs’ future use of human cues is highly malleable depending on reinforcement history (Elgier et al., 2009). All of this does not mean that breed differences (to the extent they exist) are either phylogenetic or ontogenetic – they are most likely both. We should keep this in mind in order to avoid the nature–nurture fallacy.

After the first wave of research on dogs’ understanding of human cues, the last decade has devoted work to the question of how subtle (and perhaps unintentional) human cues impact communication interactions between dogs and human (e.g., Kupan et al., 2011; Kis et al., 2012; Marshall-Pescini et al., 2012). Furthermore, researchers have attempted to find the key components or features of the human pointing gesture that contribute to dogs’ understanding of it as a communicative action. It may come as a surprise that it is still not clear whether dogs understand the communicative intent of the signaling human or whether they react only to some cuing that directs their attention to the reward. Earlier work showed that dogs are able to rely on relatively novel gestural forms of the human communicative pointing gesture and that they are able to comprehend to some extent the referential nature of human pointing (Soproni et al., 2002). However, recent advances in this research indicate that dogs do not necessarily interpret pointing informatively, that is, as simply providing information, but rather as a command, ordering them to move to a particular location. In one study, dogs ignored the human’s gesture if they had better information, and followed children’s pointing just as frequently as they followed adults’ pointing (and ignored the dishonest pointing of both), suggesting, according to the authors, that the amount of own knowledge but not the level of authority affected their behavior (Scheider et al., 2013). Both findings suggest that dogs do not see pointing as an imperative command but as an informative or referential cue. This does not mean, however, that dogs use higher levels of reasoning to understand the signal, the more parsimonious explanation is that dogs follow human pointing based on associative learning mechanisms, having learned in their individual ontogeny that the human’s pointing is often connected to rewards (e.g., Wynne et al., 2008; Dorey et al., 2010). Still, ongoing research is looking into the question of whether dogs react to human pointing gestures in acts of joint communication and shared information.

The latter account of dog’s understanding of human behavior is interesting with respect to the meanwhile hotly debated question of whether dogs, like humans (Tomasello et al., 2005), understand other individuals’ communicative intent based on some understanding of them as mental agents. Less than a decade ago, the majority of dog researchers were rather skeptical in this respect, assuming that dogs’ interpretation of referential behaviors is based on a fairly restricted set of cues (for instance, Wobber and Kaminski, 2011; Kaminski et al., 2012). They were inclined to propose non-mentalistic accounts, which they thought would be sufficient to explain dogs’ skills with human communication and enough for guiding dogs’ movements within space. Indeed, nothing more would be needed to use dogs during certain activities like hunting and herding.

Still, the area between a completely mechanistic and a completely mentalistic account is huge. At the middle ground we may see dogs being sensitive to humans having visual perspectives that are different from their own. For instance, Bräuer et al. (2004) confronted dogs with a situation in which they were forbidden to take a piece of food. Dogs stole significantly more food if they could be seen by the human, even only through a hole in the wall, showing that to some extent dogs seemed to be sensitive to the human’s visual perspective (Bräuer et al., 2004; Kaminski et al., 2009). But is this sensitivity simply a result of associatively learning to respond to direct cues (e.g., the human can be seen), or can dogs infer from indirect cues what humans can or cannot see? The results of two recent studies indicate the second possibility. In a food-stealing task dogs seem to understand that, when the food (and therefore the area around it) is illuminated, the human can see them and, therefore, they refrain from approaching and stealing the food (Kaminski et al., 2013). In the second study, dogs showed that they can understand something about a human’s perspective, because, out of two humans informing of where food was hidden, they relied on the one who could see the food hiding process (Maginnity and Grace, 2014). In this famous “Guesser–Knower task” (Povinelli et al., 1990), dogs used cues directly related to the humans’ visual access to the food, like whether their eyes were open, whether they were directed to the hiding locations, and whether the informant remained in the room during the hiding.

Very recently we replicated the second study, but added a condition in which no directly observable cues could tell the dogs who would be the knower and thus reliable informant (Catala et al., 2017). The critical control for behavior-reading, as the less demanding alternative to mind-reading, involved two informants that showed identical looking behavior during the food hiding event. However, due to their different position in the room, only one had the opportunity to see where the food was hidden by a third person. Using geometrical gaze following, dogs could infer who could possibly see the food hiding, and whom to trust. By choosing the help of the knower but ignoring the help of the guesser dogs showed perspective taking.

We still have to be careful and avoid over-interpretation. Geometrical gaze following, despite being seen to rest on a cognitively sophisticated mechanism (Fitch et al., 2010), does not require mind-reading; the recognition of mental states like beliefs, desires, and intentions. The dogs’ confidence in the informant who was in the position to see the relevant event (food hiding) might be a product of generalization from similar situations in everyday life (Udell et al., 2011). Still, even this does mean something: dogs seem to observe humans closely, form behavioral rules from this and apply them to new contexts. The reluctance of dogs to follow the looking-away person could have been learned in similar, but not identical, situations during their life in the human vicinity. In numerous cases they have seen what consequences human looking behavior has, that it is easier to communicate with humans whose eyes are visible and who look at instead of away from a target, and that they ignore things they have not seen before. It becomes obvious that living with humans puts a lot of intellectual baggage on the individual dog’s learning history. This means, on the other hand, that in order to deal with humans, dogs need opportunities to be with them, observe them, and learn from situations. Still, more research about what dogs understand about the intentions and even beliefs of humans is necessary to confirm dogs’ recent inclusion in the small circle of models of non-human perspective taking in a cooperative and hetero-specific context.

Taken together, these findings show us that dogs are sensitive to human gestures, can learn their meaning, and seem eager to cooperate. They understand gestures as imperative commands but also to some extent as informative or referential cues, engaging with humans as communicative partners. Thereby, they do not necessarily subordinate their own perspective to the human one: they take their own (well-informed) knowledge into account when given (ill-informed) commands. Especially dog breeds that have been bred for cooperative work are very good at understanding human gestures and commands. On the other hand, individual training opportunities seem important: shelter dogs for example are less successful than pet dogs at following human pointing gestures. Furthermore, the dogs’ reinforcement history shapes their understanding of human gestures. Dogs have been found to be excellent behavior-readers if given the opportunity. They are highly competent in learning about directly observable but also quite subtle behavioral, gestural, vocal, and attentional cues, which is of high adaptive value for life in the human environment. In addition to their behavior-reading competences they also seem to be sensitive to some mental states in humans. They for example seem to know that humans have visual perspectives different from their own.

#### Understanding Human Actions: How Dogs Learn Our Social Game

Dogs have impressive capacities for social learning. This competence shines through in almost all forms of social learning, including local enhancement (e.g., Mersmann et al., 2011), stimulus enhancement (e.g., Kubinyi et al., 2003), emulation (e.g., Miller et al., 2009), motor imitation (e.g., Huber et al., 2009), selective imitation (Range and Huber, 2007), and deferred imitation (e.g., Fugazza and Miklósi, 2014). They not only benefit from having the opportunity to learn from humans, they actually learn something relevant. For instance, they learn to make a detour to find food (Pongrácz et al., 2001), learn how to manipulate objects (Kubinyi et al., 2003; Pongrácz et al., 2012), and learn the direction in which a sliding door has been pushed to get some treats (Miller et al., 2009). In addition, they are able to anticipate the caregiver’s action, and as a result they synchronize their behavior with that of their caregivers (Kubinyi et al., 2003; Duranton et al., 2017). This implies that their learning is not only shaped by goal-directedness but influenced by other factors as well. This even applies to strategies that are seemingly unproductive or dysfunctional but nevertheless used by someone they observe.

Only recently it has been shown that dogs engage in what has been termed “overimitation,” the copying of unnecessary or causally irrelevant actions (Lyons et al., 2007). This peculiar form of copying was until that time considered a uniquely human capacity, which likely played a key role in why human culture can accumulate over time (Clay and Tennie, 2018). It had been assumed that humans overimitate not only for cognitive and normative reasons, but also to satisfy social motivations. They attempt to “affiliate with or be like the model” (Nielsen, 2006; Keupp et al., 2013, 393). If dogs show this behavior as well, it could highlight how deep they are enculturated in our human world because their readiness to overimitate could highlight their affiliation with closely bonded humans as a motivation for behavior.

A first study with canines provided suggestive evidence for overimitation (Johnston et al., 2017). In the test, the experimenter first established a positive relationship with the subjects by feeding them and then demonstrated how to open a puzzle box, but also performed a causally irrelevant action onto the box (moving a non-functional lever). Surprisingly, half of all tested dogs and dingoes copied both actions, although in further tests some stopped replicating the irrelevant action.

In two studies in the Clever Dog Lab in Vienna, the two actions had been separated both spatially and temporarily in order to ensure that the dogs did not confuse their causal natures (Huber et al., 2018, 2020). The causal action consisted of opening a sliding door that blocked the access to a treat; the irrelevant action involved touching colored dots that were mounted on the wall at a distance. Touching the paper sheet had no effect and was not necessary for getting the treat. Despite its irrelevance, almost half of the dogs replicated the touching action (Huber et al., 2018).

Before dogs had been tested on overimitation, several studies with great apes failed to show similar effects; they did not even show a tendency to copy the demonstrator’s actions that were not necessary to achieve a goal (e.g., Clay and Tennie, 2018). Chimpanzees, for instance, were found to act in a purely goal-directed, efficient manner (Horner and Whiten, 2005). This led Huber et al. (2018) to assume a social rather than a cognitive explanation for overimitation in dogs. Not only their ability to cooperate with, but also to learn from, humans seems to be closely related to their affiliative (e.g., Topál et al., 1998) and communicative (e.g., Miklósi et al., 1998) behaviors towards humans. Dogs seem to interpret a test situation as a form of communication or social game (Soproni et al., 2001), especially when the human experimenter uses ostensive cues (Kubinyi et al., 2003; Topál et al., 2009; Téglás et al., 2012; Wallis et al., 2015). And, like children, they attend more to those humans with whom they also had a close relationship (Horn et al., 2013).

In a follow-up study, we tested the hypothesis that dogs are more inclined to copy irrelevant actions if shown by the affiliated caregiver rather than by an unfamiliar person. By faithfully replicating Huber et al. (2018), using the identical methods and procedures, but only substituting an unfamiliar person for the dog owner as the demonstrator, we found a measurable decrease in the number of dogs that copied the irrelevant action (Huber et al., 2020). This finding thus confirmed our hypothesis that overimitation is facilitated by the affiliative relationship between the human demonstrator and the imitating dog, satisfying social motivations. Family dogs may repeat the actions of the human partner either because they want to please their caregiver or because they are inclined to obey by following tacit commands. While the first is clearly a positive characteristic of the dog–human relationship, the second one is ambiguous, although the two are linked. However, it is also possible – although difficult to prove – that the dogs overimitate because they want to be part of our social game, meaning that they want to be included in the social interaction that is happening. This interpretation is based on the assumption that they could have a social motivation to affiliate with the model and want to “be like the model” – as has been proposed in the case of humans to explain their readiness to overimitate (Nielsen, 2006; Keupp et al., 2013, 393). Here, to “be like the other” could mean that the dogs want to *behave* like the other and *be with* the other. This explanation is compatible with the existence of an urge to please the caregiver or an inclination to obey. The intention to preserve and foster the bond between human and dog, however, may be in itself a motivation behind this behavior. A dog might furthermore trust her caregiver in such a profound way that she sticks to whatever the caregiver proposes, at least for a while. Thus, it takes her some time to detach from the caregiver’s irrelevant strategy and come up with a more efficient one herself. In a team that is usually built on trust and affiliation this makes sense as a social strategy. It is surely difficult to test for such explanations based on trust or affiliation, but that should not be a reason to rule them out right from the beginning. Complex social motivations in animals are clearly getting increased attention from empirical research lately. Disentangling the affiliative bonds between dogs and their caregivers, their scope and meaning, is one of the big challenges we face.

Cumulating evidence suggests that the relationship between companion dogs and their human caregivers bears a remarkable resemblance to the parent-infant attachment bond (Archer, 1997; Topál et al., 1998; Gácsi et al., 2001; Prato-Previde et al., 2003; Hare and Tomasello, 2005; Prato-Previde and Valsecchi, 2014). This affiliative bond changes dogs’ behavior in multiple ways. It enables dogs to engage their caregiver’s caregiving system, and it affects the way the dog explores objects and performs in cognitive tasks (Horn et al., 2012, 2013). Like in children, the bond not only changes the dog’s general attitudes towards humans, it is also selective. For instance, dogs pay more attention to the actions of their caregivers than to the actions of other familiar humans (Horn et al., 2013). And again, like in the case of the human parent–infant bond, the quality of the bond has strong influences on all these changes just mentioned (Myers, 1984; Ainsworth, 1989).

Taken together, these findings show that dogs pay close attention, not only to the emotions and gestures of humans, but also to their actions. They even overimitate, thus showing a specific copying style that is believed to be a crucial feature of cumulative human culture. Overimitation in dogs is another strong sign for how deeply they attend to humans, especially to those with whom they have close relationships. The bond (which is selective) and the quality of the bond are of great importance for dogs’ general attitude towards humans and their behavioral performance. This can be nicely seen in family dogs interacting with their caregivers. Why dogs attend so closely to the behavior of their caregivers can be explained by different reasons: they surely want to please them and are inclined to obey them. However, they might also understand themselves as partners in our social interactions and are part in our social game. Bonding and affiliation are to be understood as motivations for social interaction. Humans make ample use of the dogs’ readiness to understand their actions: dogs are trained in many different ways and for many different reasons, including agility training, obedience training, and other forms of special-purpose training, in which a precise following of the trainer’s behavior is the rule (Clark and Boyer, 1993).

#### Moral Emotions? From Biology to Philosophy

Dogs are deeply entrenched in interactions with humans, for which they are equipped with outstanding skills to understand human emotions, gestures, and actions. They form cooperative teams with us (e.g., as assistance, rescue, or herding dogs), they engage with us as communicative partners, and they have been enculturated in our society and are clearly part of our social game. Bonds between humans and dogs can be very intense and even resemble parent–infant attachment bonds. It seems to be this specific relationship of shared understanding and close affiliation that is at the heart of the view that dogs are indeed humans’ best friend.

Besides the capacities we mentioned there might be other, social and cognitive abilities in dogs, some of which we do not know much about so far. Possible candidates for such capacities could be empathy, guilt, or jealousy.

Empathy can be understood, following de Waal’s Russian doll model, as an umbrella term that covers all those ways in which one can be affected by others’ emotions. The capacity for emotional contagion lies at its core, and outer layers of this “Russian doll” can incorporate more cognitively demanding capacities, such as theory of mind, perspective-taking, and sympathetic concern (e.g., de Waal, 2008). While the available evidence suggests that dogs are capable of emotional contagion (Sümegi et al., 2014; Yong and Ruffman, 2014; Palagi et al., 2015; Quervel-Chaumette et al., 2016; Huber et al., 2017; Bourg et al., 2020), researchers are still on the look-out for empathy-based complex behavior. First results indicate, for example, that there is “empathetically-motivated prosocial helping in dogs” and that dogs “are most likely to provide help to a human in need if they are able to focus on the human’s need instead of their own personal distress” (Sanford et al., 2018, 386). However, such results stand against mixed evidence on dogs’ helping behavior and against the need to clarify the underlying emotions and motivations (see e.g., Macpherson and Roberts, 2006, or the discussions in Sanford et al., 2018 and Adriaense et al., 2020). Because empathy could motivate moral behavior like helping, philosophers of animal minds and animal ethicists discuss it as a moral emotion that animals could possess (Rowlands, 2012; Monsó, 2015, 2017; Monsó et al., 2018; Benz-Schwarzburg et al., 2019).

Two other interesting candidates for moral motivations that could also shape the social interactions and relationships between dogs and humans are guilt (see e.g., Tangney et al., 2007; Prinz and Nichols, 2010) and jealousy (see e.g., Fredericks, 2012; Kristjánsson, 2015). However, the evidence here is ambiguous or non-existent. There is to our knowledge not a single paper that provides strong empirical evidence of dogs feeling guilty. On the contrary, preliminary evidence suggests that dogs are not capable of guilt, despite many owners’ perception to the contrary (Horowitz, 2009; Hecht et al., 2012; Ostojić et al., 2015). Owners indeed often interpret their dogs’ behavior as guilt (Hecht et al., 2012), something that can be ethically problematic: “Failure to read these gestures for what they are, or even worse, misinterpreting gestures of appeasement as a sign of the dog feeling guilty, are likely to lead to inappropriate responses on the part of the human in the situation and hence lead to escalation of the behavior resulting in lunging, snapping, and/or biting” (Mills et al., 2014). The case of jealousy is similar. We are just starting to investigate this emotion in dogs and face a limited body of research results. Interesting insights were reported by Harris and Prouvost (2014) who believe that at least some “primordial” form of jealousy, which we know from human infants, occurs in dogs as well, or from Cook et al. (2018) who investigate jealousy in dogs *via* fMRI methods. However, the results are heavily debated (see e.g., Vonk, 2018).

Interest in the named abilities in animals is rising among philosophers. This is at least partly because the presence of moral emotions in animals would mean that animals qualify as moral subjects, that is, individuals who sometimes behave on the basis of moral motivations (Rowlands, 2012). Moral emotions thus mark a minimal form of animal morality. This is ethically important. Indeed, it has been argued that minimal morality gives us a reason to owe these animals a special moral consideration, one that goes beyond the welfare approach that we so often use to evaluate our treatment of animals, be it pigs or dogs, cows, or any other non-human species (Monsó et al., 2018; Nawroth et al., 2019). If animals are moral subjects, profound ethical implications could follow, for example in the shape of animal rights (Rowlands, 2012), something we have already seen defended in ethical debates surrounding great apes (see e.g., Andrews et al., 2018). However, capacities such as empathy, guilt, or jealousy are very difficult to define conceptually (from a philosophical as well as a biological perspective). This is the case even if researchers pay much attention to them, as can be seen in the case of empathy, of which it has been said that “there are probably nearly as many definitions … as people working on the topic” (de Vignemont and Singer, 2006, 435). Adriaense et al. (2020, 62) conclude that we still face the challenge here of “closing the gap between theoretical concepts and empirical evidence.” The emotions of guilt and jealousy face similar definitional problems that will surface more and more when research into them proceeds.

Research into moral emotions and other social phenomena in dogs will surely add to our understanding of their perception and behavior in the future. Perhaps we should err on the side of caution and assume that dogs are indeed moral subjects. However, based on the current state of the evidence we cannot make conclusive claims, yet. In addition, the discussion still needs conceptual input, and so we call here for interdisciplinary research on this topic. While embarking on this challenge, we should constantly re-evaluate how far our ethical thinking leads us with reference to less controversial research results, as well as maintain an open mind towards challenging inherited definitions of different capacities when there are good conceptual reasons to do so. After all, the philosophical debate on social capacities in animals increasingly leans towards de-intellectualized accounts of such abilities in animals, including moral abilities (Rowlands, 2012; Monsó, 2015) and towards an investigation into their ethical relevance (Monsó et al., 2018; Benz-Schwarzburg et al., 2019). In any case, our point in the following section is that we already face good reasons to arrive at a more profound ethical consideration of dogs than we often grant them. We will settle with the kind of ethical implications that we can derive safely by focusing on the kind of research results summarized in sections “Understanding Human Emotions: How Dogs Read Our Faces and Listen to Our Voices, Understanding Human Gestures: How Dogs Learn to Cooperate, and Understanding Human Actions: How Dogs Learn Our Social Game”. We believe that the mentioned capacities suffice to argue that dogs have a profound understanding of human gestures, actions and emotions. They clearly bond with us and enter into relationships of mutual understanding and meaningful interaction. Such relationships have repeatedly been described as characterized by attachment and close bonds. Let us build an ethical argument on that.

## Characterizing the Human–Dog Relationship: Ethical Perspectives

Until now, we have very much emphasized a positive outlook on the human–dog relationship. It would be a one-eyed view if we would only mention the obviously positive aspects. For any ethical discussion concerning pet dogs we need to understand that, on top of the affiliative motive, the behavior of these animals vis-à-vis their caregiver is also determined by their dependency on us and thus on educational and normative influences that need to be examined carefully. In the household, humans educate the dog regarding what to do and what not to do, involving actions that are far from causally transparent, and may be purely arbitrary or – even less positively – exclusively human-centered. In dog training, for example, a precise following of the trainer’s orders, commands, or behavior is the rule – and in fact expected from the dog, independently of the bonds at play, no matter what the dog’s own preferences for some humans over others are, and irrespective of the dog’s own intentions and desires. Are not there a lot of ethical challenges involved in the fact that dogs are so much part of the human world?

In what follows, we will engage in a brief ethical discussion of the human-dog relationship. As a necessary first step, we will characterize the human-dog relationship as one in which there is a necessary power imbalance, where one of the partners is always more powerful than the other. Following that, we will give an overview of the ethical responsibilities that arise out of this inequality when we consider it in connection to how dogs perceive us and to the pervasive influence that we can have on their character and capabilities. The owner or caregiver has certain duties, we will argue, that go beyond ensuring an adequate welfare of their pet.

### The Human-Dog Relationship as a Power-Relation

Ethicists have argued that the human–dog relationship oscillates between two extremes: dogs, like other companion animals, are at the same time “pampered” and “enslaved,” something that constitutes a “moral dilemma” (Irvine, 2004). “Enslaved” in this instance is to be understood as a philosophical term, coming from an ethical approach that departs from the fact that companion animals exist for human purposes and are defined by the law as our property (Irvine, 2004, 5). We can add aspects of dominance, ranging from a restriction in personal freedom (covering all aspects of a dog’s life, like feeding regimes, mating choices, or neutering policies) to forms of labor (like the use of dogs as sheep-herding, guiding, sniffing or rescuing staff). Most importantly, it is questionable whether dogs give in any form their free and informed consent to fulfill the tasks we assign to them. Dogs are clearly capable of cooperating with humans (skills-wise) and often happily seem to do so. But freedom (even in a minimal sense) is about opportunities and choices, and how much of these do they have? As we are talking about an animal that is very much dependent on her caregiver’s choices and who is being purposefully bred as well as (often quite heavily) trained to fulfill certain human-oriented tasks, the question seems warranted (Cochrane, 2009, 2012; Schmidt, 2015).

Thereby, it seems possible, and even morally desirable, to grant an animal more choices and thus more freedom. Yeates (2015, 168) identifies a range of situations where we should from a normative perspective respect the animal’s choice. These are for example situations in which we ourselves lack “accurate knowledge of the animal’s subjective experiences,” or in which we do “not know what will lead to desirable experiences or allow for the avoidance of undesirable ones,” when we are “biased” or “less aware of the animal’s specific situation.” He argues, furthermore, that we should better turn to respect the animal’s choice when we ourselves “cannot appreciate all elements comprehensively, including considering any value to the animal being allowed to make and implement a choice, such as where a lack of control or liberty would be unpleasant or where an animal would usefully learn from the process of choice-making.” Such an approach ultimately aims to reduce the power hierarchy and “set up situations that empower animals” to make their own choices.

Up to now, the high amount of paternalism and training involved in the human–dog relationship gives rise to a clear power relation. For sure, more and more trainers adopt training methods that turn away from a behavioristic understanding and work in a scientifically informed manner. But the many different perspectives on suitable training methods and the many noncertified methods and noncertified institutions in the dog training business lead to much diversity in the field. Thus, even though the field has moved forward in the past few years, it seems difficult to assess how scientifically informed the majority of trainers (let alone owners) actually treats and trains their dogs. Also, some dog trainers with massive public outreach even add on the mentioned questionable understanding by arguing that all dog training is ultimately about teaching the dog that the human is pack leader. Cesar Milan, one of the most influential and controversial dog trainers, describes “Pack Leadership” as a core principle of his training strategy, to be applied in the following way: “Establish your position as pack leader by asking your dog to work. Take him on a walk before you feed him. And just as you do not give affection unless your dog is in a calm-submissive state, do not give food until your dog acts calm and submissive” (Milan, 2019). Still, even without such an idea of discipline and submission, other forms of dog training based on purely positive reinforcement also resort to methods that heavily impact on the dog’s will, her choices, preferences, and intentions. Some methods tie almost all feeding to training steps by reinforcing every positive behavior with food, sometimes while putting the dog otherwise on food deprivation. Lindsay describes in his *Handbook of Applied Dog Behavior and Training* that training only works if the animal is “in a state of need” that can be satisfied only after the dog behaves in a “predetermined way.” Therefore, “combining food deprivation together with the presentation of special treats produces the best training results. The term deprivation means scheduling training sessions before meals rather than after them. The meal itself can be given to reinforce the overall training session as a sort of jackpot” (Lindsay, 2000, 249).

We have come across a substantial reinterpretation of affection as something that is not given to the dog “unless the dog is in a calm-submissive state” in Milan’s (2019) training procedure and another substantial reinterpretation of feeding the dog in the sense that meals become a “sort of jackpot” in classical, modern reinforcement training. These narratives are normatively relevant because they show the tight entanglement of power, predetermination, and submission in dog training, expressed by a language in which dogs “work” for us. No matter the method, all training ultimately educates the dogs into a human world with the aim that they function properly, that is, according to valued and dis-valued behavior in this setting: they are not supposed to chew on our furniture, pee on our carpet, or chase the neighbor’s cat. Spaces where a dog can, for example, run free without a muzzle or leash and interact with other dogs are clearly restricted as well as rare, at least in urban settings, where numbers of dogs have been increasing dramatically over the past decades, standing currently at well over 60 million in the United States alone (American Pet Products Manufacturers Association, 2020).

We are aware that this understanding draws a rather sobering picture of the often romanticized human-dog relationship. However, pet keeping is not a given or simply a result of a natural affinity between humans and animals. It is a historically contingent practice that has also been circumscribed by social class and gender constructs (Irvine, 2004, 19). This is a sociological point that links with ethical and biological perspectives: like all our relationships with companion animals, the human-dog relationship depends on how we define animals, and for that our knowledge about their abilities and needs seems crucial. For sure, it is also crucial how ready we are to take their perspective into consideration. For this, questions of power and hierarchy are relevant.

So let us start from the premise that the human-dog relationship can be described as a human-dominated power relation in which dogs often have little choices and humans perceive themselves on a spectrum between guardians and leader of the pack. Given this power relation at place, and given a generalized lack of awareness of the latest research on dog social cognition, humans tend to interpret communicational misunderstandings as problems of the dog (e.g., in the sense of non-obedience). They consequently tend to interpret the behavioral reactions of the dog to such miscommunication not as a result of miscommunication (for which they themselves are also co-responsible) but again as a problem of the dog, who is, for example, claimed to be aggressive. Humans need to take responsibility here. We are left with the necessity to better understand how dogs perceive us and what they are capable of. Our summary of the socio-cognitive abilities of dogs only shows the tip of the iceberg of what these animals can do. We should not forget that they are quite different from ourselves with respect to their perceptual repertoire: humans, other than dogs,^3^ rely much more on vision, are relatively insensitive to odors, and so forth. Taking our visual perceptions, our facial expressions, and our emotions and actions into account to the extent dogs obviously do, renders their social life rather complicated. Living in a human’s world can thus be very demanding for dogs and some dogs might be overwhelmed. It is our responsibility to gain awareness of the challenges we face them with.

In addition, we need to deepen our understanding of the kind of relationship we offer them and the power relations characterizing it. Here too, gaining awareness means shifting the focus from the dog to the human, and consequently taking responsibility. We need to arrive at a better understanding of the range of concrete duties dog owners have. In what follows, we will argue that humans are to a large degree responsible for who their dog turns out to be and that they have a duty to ensure her adequate flourishing. Not only this, the characteristics of the human-dog relationship point to a propensity towards trust on behalf of the dog, and consequently entail a duty not to betray that trust.

### The Duties of Dog Caregivers

In animal ethics, there is a generalized agreement that humans have negative duties towards (at least some) animals. Negative duties refer to duties not to cause unjustified harm, a position that can be defended from a number of ethical theories, including utilitarianism (Singer, 2009), deontology (Regan, 2004), and virtue ethics (Hursthouse, 2011). However, negative duties do not exhaust all that morality demands from us. In human-human relationships, we are also often required to assist someone in need, even if we are not responsible for their harm. For instance, if we witness someone falling onto the train tracks at an underground station, we are morally required to do our best to save them, even though their peril is not our own fault. These are known as positive duties. In those cases, in which there is a pre-existing special relationship, these positive duties are even stronger. Parents are not only required not to harm their children and to assist them when they are in need, they are also required to do all that is in their power to ensure that they have a good life. This means providing them with food and healthcare, but also ensuring that they receive a proper education, that they have opportunities for exercising their creativity and making friends, that they feel loved and cared for, and so on. In short, that they flourish as the sort of beings they are. Rowlands (2012) considers that this treatment is owed as a matter of respect: “to respect an individual is, fundamentally, to respect it as the kind of individual it is” (Rowlands, 2012, 249). If, indeed, the dog-human relationship entails forms of attachment that resemble our bonds with human children, the question then arises: what would respecting our dogs as the kinds of beings they are look like?

Palmer (2010) has argued that when considering the duties that we owe to other animals, we cannot follow a one-size-fits-all logic, even in those cases where different species have similar cognitive capacities. She argues that the surrounding context, the history, and the pre-existing relation are fundamental in determining the kinds of duties that we owe to a particular animal. With regards to those animals who live independently from us in the wild, we only have negative duties not to harm them. In contrast, those animals with whom we have some sort of special relationship will, in addition, generate positive duties. If we consider the case of dogs, this is clearly going to be one of the most demanding human–animal relations from a moral perspective. As we have already discussed, dogs are the oldest domesticated species. This history has generated a very high degree of vulnerability and dependency in those dogs that live in our households. They depend on us for food, shelter, and medical care. Indeed, they depend on us for sheer survival. As we have seen, dogs also have a highly malleable nature and we can shape their character to a large degree. Dogs play very little part in choosing their caregiver, and still the person they end up with will have a profound influence on their life and on the sort of individual they turn out to be. So, they also depend on us to a much deeper level. This, coupled with the aforementioned power relation, generates positive duties that go beyond simply ensuring that the dogs in our household have a good welfare.

We are responsible for our dogs’ lives from beginning to end, and this means that we will have an immense causal influence on the quality that their life ultimately has. This generates a duty to ensure that our dogs lead a good life. But what does it mean for a life to be good? Different philosophical traditions have offered different answers to this question (for an overview of these different theories see Crisp, 2017). From the perspective of a common theory known as hedonism, a good life is one in which there is, overall, more positive subjective experiences than negative subjective experiences. For a dog this might mean a life in which she is in general happy and has very few painful or fearful experiences. From the perspective of desire-satisfaction theories, in contrast, a life is good if the individual’s most important desires are fulfilled. For a dog, this could mean a life in which she gets to do all the things that she really cares about. We believe that neither of these two options gives a satisfactory account of what it would mean for a dog to have a good life.

It is easy to see why the desire-satisfaction account of a good life is not adequate, at least in the case of dogs. This is due to the mismatch between their biological roots as wolves and the fact that they have been domesticated. This history has led to a situation in which, firstly, not all the desires that dogs have are actually good for them. For instance, many dogs, if let by their caregivers, will eat much more than they actually need, and consequently develop different health problems in the long run. The tendency to eat more than needed might be good for a carnivore who lives in the wild and does not get to eat very often, but for a pet in a household with unlimited access to food, it can significantly worsen her quality of life. Secondly, it is not just important to determine what dogs desire, but also what are the reasons behind those desires. As we saw before, dogs are very often eager to cooperate with humans, but it is difficult to see what the exact motivation behind this eagerness is: Is it the expectation of a reward? Is it fear of punishment?, or Is it a desire to please the caregiver or a desire to be part of the social game? The history of domestication has also led to dogs being shaped to be eager to cooperate with us. In this sense, many of their desires are the result of a process of selective breeding that could be comparable to a process of indoctrination in humans. Therefore, from the fact that a dog has a desire, the conclusion that it is good to satisfy this desire does not automatically follow.

Desire-satisfaction theories thus cannot provide us with a satisfactory account of what it means for a dog to lead a good life. But what about hedonism? Surely a life in which a dog is overall happy is a good life for that dog? We believe that hedonism, just like desire-satisfaction theories, captures an important aspect of what it means to lead a good life, but cannot give us the full story. In philosophical terms, having more positive than negative subjective experiences throughout one’s lifetime is a necessary but not sufficient condition for a good life. Imagine a dog, we can call her Frida, whose caregiver decides to keep her inside the house her whole life to protect her from possible dangers and fearful stimuli she might encounter outside. Frida is provided with an adequate diet, a comfortable bed in which she can rest, and enough toys to keep her entertained. The extremely controlled environment she is kept in ensures that she very rarely experiences any accidents or illnesses, stress or pain. If we look at Frida’s life as a whole, we will see that she is extremely pampered, to say it in Irvine’s words, and overall happy. But is this a good life?

We believe that Frida’s life, while certainly far from terrible, is not a good life. This has to do with the fact that, by not being allowed to encounter challenges, to interact with con- and heterospecifics, and to explore the outside world, Frida is prevented from flourishing as the type of being that she is. As we saw in the previous sections, dogs have many amazing socio-cognitive skills but these are largely dependent on how we have shaped them during domestication and what they learn from interactions with humans during ontogeny. We believe that caregivers have a positive duty to ensure that these capabilities can develop, not only so that the animals can better cope with the challenges they might encounter in their lives, but also because it is a good thing for them to be allowed to flourish as the type of being they are, an idea that can be captured, for instance, using the capabilities approach (Nussbaum, 2007; Monsó et al., 2018).^4^ Allowing for the dogs in our care to develop their socio-cognitive skills also enables them to have a life that is more meaningful. According to Purves and Delon (2018), animals’ lives acquire meaning when they are allowed to exercise their agency and use it to bring valuable states of affairs to the world. These valuable states of affairs range from relatively simple endeavors like rearing their young or establishing friendships, to more demanding behaviors like rescuing a human in need (which recent research shows dogs are capable of; Bourg et al., 2020). A dog who is allowed to flourish to her full capacity is more likely to lead a meaningful life, which will in turn be a better life.

In addition to the duty to ensure the flourishing of the dogs under our care, there is also an additional duty that emerges from the special relationship that we have towards dogs, and from the specific way in which dogs perceive us. In our review of the empirical evidence regarding dogs’ perception of humans, we have highlighted the special characteristics of the dog–human bond. Dogs are not only eager to cooperate with us; they are also attuned to us like no other species. Their tendency to overimitate humans, for instance, points to a perception of us as important social partners. We know that dogs can recognize individual humans and they are also significantly less fearful of us than are their wild ancestors. All of this points to the ease with which a relationship of trust among dogs and their caregivers can emerge. Placing your trust in another allows for significant social bonds to be built, but it also means that one is made more vulnerable. The moral importance of this was captured by Cooke, who wrote that “[i]n trusting another, we give them power over us, power to set back our projects, exploit us, and make us vulnerable not just to them, but to others also” (Cooke, 2019, 188)^5^. The trust that dogs place in us is no coincidence; instead it is a result of the process of domestication of which we are at least partly responsible as well as a result of what they learn in interactions with us during their lives. Humans thus have a duty to live up to this trust (see similarly Hens, 2008), to ensure that our dogs’ needs are met, and that they are not placed in a situation where it would be warranted for them to feel betrayed. To paraphrase Cooke (2019, 198), humans have a duty to act in ways that make them worthy of the trust that dogs place in them. For this duty to exist, it is not necessary for dogs to possess a cognitively complex form of trust for which we do not have any empirical evidence, yet. Our argument is that the way dogs engage with us evidences a trusting relationship that gives rise to duties on our side (not on theirs). For the kind of trust we are after we do not need the dog as a moral agent to fully understand what trust is in a normative sense, nor do we need the dog to understand duties on her side. Dogs’ capacity to enter into such relationships with us is independent of the question of whether they have (in addition) the sort of capacity for full-blown moral judgment that orthodox frameworks of moral agency require, or even a simple explicit motivation to trust their owner (which could make them a moral subject in Rowland’s sense). At least the former, intellectually demanding forms of trust might be tied to other complex abilities, such as a theory of mind. Our point is humbler here but still of profound relevance: the kind of trust we identify in the human-dog relationship becomes an ethical signpost in the light of the dog’s dependency on her caretaker.

## Conclusion

Dogs have indeed special skills to understand and interact with humans due to the evolutionary history and domestication of the species and due to complex competences acquired by individual and social learning. We see accumulating evidence of their understanding of human emotions, gestures, and actions and of how much they are thus part of human culture and our social game. Bonds between dogs and humans are selective, intense, and vary in quality. Affiliation plays a motivational role in dog behavior and shapes the dogs’ attitudes as well as their interaction with humans. All of this, however, has to be seen in the light of a comprehensive characterization of the human-dog relationship, which is a socially constructed practice with clear power relations. We have argued that the human-dog relationship is a dominance relationship where humans are usually in command of power. If caregivers are unaware about how much their dogs pay attention to subtle communicative cues and how much they understand about as well as attend to their caregivers’ emotions, gestures, and actions, a range of conflicts can arise. Instead we should invest into building relationships of trust with dogs that live up to ideas of companionship.

Irvine (2004) arrived at the conclusion that “relationships between humans and animals have depended on how a given society defines animals and what it means to associate with them”. She argues that “what we currently know about animals demands wrestling with the moral implications of keeping them as pets” (Irvine, 2004, 5). We have been following this critical view of pet keeping in general and dog keeping in specific, because it could serve as a helpful heuristic to map out problems that are often overlooked, specifically problems that point beyond welfare towards other normative concepts. Sixteen years after Irvine’s paper we face a substantial amount of new research results on dog social cognition which we have summarized in this paper and which we need to take into account when debating the human-dog relationship today.

From what we have discussed we gain a better understanding of a main characteristic of the human-dog relationship that lies in its dichotomy between special attachment as well as special understanding on the one hand and the instrumentalization of dogs on the other hand. Against this backdrop, a meaningful social interaction between dogs and caregivers remains a fragile construct. In order to treat dogs in the way that morality requires of us, it is paramount that we bear in mind the spectrum of positive duties that this relationship engenders, including the duty to live up to the trust that dogs place in us.

## Author Contributions

All authors listed have made a substantial, direct and intellectual contribution to the work, and approved it for publication.

### Conflict of Interest

The authors declare that the research was conducted in the absence of any commercial or financial relationships that could be construed as a potential conflict of interest.

The handling editor declared a past co-authorship with one of the authors JB-S.

## Footnotes

^1^There is the possibility that dogs “domesticated themselves” to exploit a niche associated with the anthropogenic environment. However, given humans’ interest in domesticating (and then breeding and keeping) all sorts of species for their purposes we find it hard to believe that in dogs it was a one-sided process with all agency ranging on the dog’s side.

^2^We do not want to go into possible reasons for this, however, it needs to be noted that a whole range of different reasons for this could exist. After all, a household is a very different environment than a shelter. It is possible that it shapes the dog’s cognitive abilities. Likewise, their abilities (or perceived lack of abilities) might already have been a reason why their caregivers abandoned them.

^3^Dog’s sensitivity to odors allows humans to train them as sniffer dogs for all sorts of purposes, from finding substances, like marihuana or explosives, to discovering injured persons after earthquakes, from detecting illnesses, like cancer, to helping out in species conservation (see Fischer-Tenhagen et al., 2017).

^4^One could object that the fact that dogs share an environment with us gives us a reason to restrict their freedom. However, it is important to remember that dogs did not choose to share an environment with us. If we were to hold a human captive in our household, it would surely be very problematic to claim that this ‘shared environment’ gives us a reason to restrict her freedom. On the contrary, the existence of a shared environment gives us a reason to respect others’ freedom and interests, as has been long defended within social contract theory.

^5^Note that Cooke’s theory does not require any sort of cognitive notion of trust as a capacity dogs have. Instead, we would argue, trust emerges as a disposition in dogs, as a result of their phylogenetic and ontogenetic makeup and it is necessary to enter into a relationship of mutual understanding and social dependency.
